# Supplementary material for: Dynamic transcriptomic profiles of zebrafish gills in response to zinc supplementation
Source: BMC Genomics. 2010 Oct 11;11:553. doi: 10.1186/1471-2164-11-553 (PMC3091702; doi:10.1186/1471-2164-11-553)
Supplement: Additional file 2 — Interactive Direct Interaction Network representing the molecular interactions between zinc, copper, iron, calcium and proteins encoded by transcripts changed by zinc supplementation. Mini web-site containing index.html and hyperlinked pages in subdirectory describing a Direct Interaction Network automatically generated based on curated interactions contained within the proprietary PathwayArchitect database. Ovals represent proteins and the circles symbolize metal ions. Objects are coloured by their abundance in zebrafish at the time-point they were significantly different from the control is a scale from -4 fold (dark green) to +4 fold (dark red). Where significant differences were found at more than one time-point, the colour overlay shows expression at the first instance. Dark blue squares denote 'binding', and light blue squares 'expression'; green squares stand for 'regulation', green diamonds for 'metabolism', and green circles for 'promoter binding'. Arrow heads indicate directionality of the interaction where annotated. All nodes and edges can be further interrogated by selecting the relative area of the image. [file 1471-2164-11-553-S2.zip › PathwayArchitect Zn xs DIN/478316.html]

# BINDING:

|  |  |
| --- | --- |
| Type | BINDING |
| Effect | None |


---

|  |  |
| --- | --- |
| Score | 0 |


---

|  |  |
| --- | --- |
| Reference Count | 3 |


---

|  |  |
| --- | --- |
| Mechanism | Unknown |


---

|  |  |
| --- | --- |
| Reference:0 || Sentence | "UVA by itself induced an activation of ERK activity, and a stimulation of AP1, NFkappaB, and STAT1 binding activity." |
| PMID | 12633740 |
| Year | 2003 |
| Species | Human |
| Journal | Free Radic Biol Med |
| RefScore | 1 |
| Source | PArchNLP |
  |
|  |  |


---

|  |  |
| --- | --- |
 Reference:1 || Sentence | "In the presence of the ERK inhibitor, binding of the AP-1 complex and of STAT1 to the related regulatory elements was inhibited." |
| PMID | 10498647 |
| Year | 1999 |
| Species | Human |
|  | Rat |
| Journal | Hepatology |
| RefScore | 1 |
| Source | PArchNLP |
  ||  |  |


---

|  |  |
| --- | --- |
 Reference:2 || Sentence | "The human iNOS promoter contains consensus sequences for the binding of transcription factors, including interferon-gamma (IFN-gamma) regulatory factor-1 (IRF-1) binding to interferon-stimulated responsive element (ISRE), signal transducer and activator of transcription (STAT) binding to gamma-activation site (GAS), nuclear factor-kappaB (NF-kappaB), activator protein-1 (AP-1), and CCAAT/enhancer-binding protein beta (C/EBPbeta); therefore, we investigated the effect of gemfibrozil on the activation of these transcription factors." |
| PMID | 12244038 |
| Year | 2002 |
| Species | Human |
| Journal | J Biol Chem |
| RefScore | 1 |
| Source | PArchNLP |
  |


---

|  |  |
| --- | --- |
